# Interferometric control of the absorption in optical patch antennas

**DOI:** 10.1038/s41598-017-03064-6

**Published:** 2017-06-07

**Authors:** Caroline Lemaître, Emmanuel Centeno, Antoine Moreau

**Affiliations:** Université Clermont Auvergne, CNRS, Institut Pascal, F-63000 Clermont-Ferrand, France

## Abstract

Optical patch nano-antennas possess unique absorption, field enhancement and concentration capabilities – but their crosssection, as well as their response outside of normal incidence are not well understood. Here we explain the large cross-section by considering that each patch nanoantenna is a cavity excited from both sides. Such a simple physical picture allows to fully understand the influence of the angle of incidence – that odd resonances have a very high absorption cross-section which decreases when the incidence angle increases, while even resonances cannot be excited in normal incidence. A direct application would be to use these structures as an optical nanometric set-square.

## Introduction

Gap-plasmon resonators are a new kind of optical metallic resonator^1^, that has emerged as particularly promising^2–6^, from a fundamental point of view^7–9^ as well as for all the applications that could be based on these structures given their extraordinary efficiency at concentrating light^2, 10^. It has actually been suggested that they could be used as cheap but very efficient selective absorbers^11, 12^, as sensors^13–15^, as well as for designing reflection metasurfaces providing an almost complete control of light^16^. Finally, the light confinement associated to their resonances is so high that an unprecedentedly high Purcell effect can be obtained using this kind of optical patch antennas^17–20^.

These resonators are literally cavities for a peculiar plasmonic mode called a gap-plasmon^1^ which presents a very large effective index. This property alone explains how deeply sub-wavelength the resonators can be made, which in turn partly explains their efficiency: the absorption cross section of a single patch can be actually as high as 30 times its geometrical cross-section^11^. The modes that can be excited in these cavities have already been studied^2, 21, 22^, and their importance largely underlined^23^. However, the influence of the incidence angle, despite its practical importance for sensing^14, 24^ for instance, is not well understood^25^ - and their extraordinary absorption cross-section hasn’t yet received a full explanation.

Here we show that the influence of the incidence angle can be accurately taken into account if a single gap-plasmon resonator is modeled as a cavity excited from both ends, making the structure a clear example of interferometrically controlled absorption^26^, or of a coherent (perfect) absorber^27^. We give analytic formula for the losses induced by the resonances inside the cavity and link it to the incidence angle. Such a description allows to understand why the absorption decreases when the incidence angle increases^25^ and explains the extraordinary absorption cross-section of the patch antenna compared to other kinds of gap-plasmon resonators^9, 28^.

## An analytic model for the absorption of a gap-plasmon resonator

**Figure 1:**
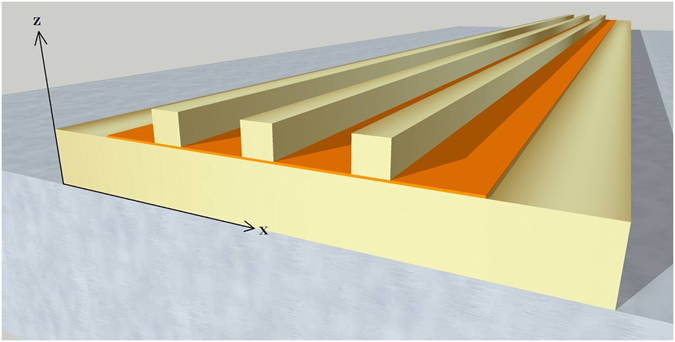
A 3D rendering of a selectively absorbing plasmonic metasurface based on gap-plasmon resonators: regularly spaced metallic nanorods coupled to a metallic substrate and separated from it by a nanometric dielectric layer.

A selectively absorbing metasurface^25^ is typically constituted of periodically or randomly distributed patches separated from a metallic film by a thin dielectric material, as shown on Fig. 1. Here we will consider silver for all the metallic parts^29^. The efficiency of the device benefits from the capacity of each patch to funnel light into the gap, so that when the resonators are close enough to each other, they are able to absorb all the incoming light and constitute a perfect absorber^12^. Most of the structures that are fabricated consist in rectangular patches coupled to a film instead of the nanorods shown Fig. 1, but is largely admitted now that the physical behavior of both structures is the same and that the difference, for the fundamental modes, essentially reduces to a shift of the spectral position of the resonances^11^. For higher order modes, although they may be more complex^22^, the fact that some are excited only for non-normal incidence^14^ is perfectly in agreement with what we expose here for 2D structures.

We must underline that when the structure is periodic, surface plasmons are often excited^4^, and can be responsible for a very large absorption in very peculiar conditions. Such a mechanism is not present in disordered structures, where the surface plasmons are excited^20^ and absorb a non-negligible part of the energy but play a lesser role than when periodicity enhances their excitation. Here we will concentrate on the resonance of a single resonator, which is angularly and spectrally broader, is more efficient, does not depend on the periodicity and explains the response of disordered structures^12^.

**Figure 2:**
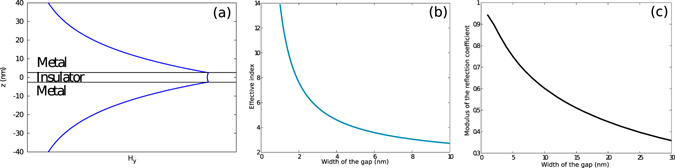
(**a**) Profile of the magnetic field associated with the gap-plasmon propagating along the *x* axis. (**b**) Effective index $\Re (\alpha )/{k}_{0}$ of the gap-plasmon as a function of the gap width *d*. (**c**) Modulus of the reflection coefficient at the edge of the cavity as a function of the gap width *d*.

In the thin layer sandwiched between the patch and the metallic film, light propagates under the form of a gap-plasmon along the *x* axis, the only propagating mode of a metal-dielectric-metal waveguide. This mode exists for *p* polarization only, with a magnetic field along the *y* axis. Solving Maxwell’s equations a looking for a guided mode with a pulsation *ω* and a wavevector *k*_*x*_ in a metal-dielectric-metal configuration actually yields a magnetic field that can be written

1 $${H}_{y}(x,z)=P(z)\,{e}^{i({k}_{x}x-\omega t)}$$

with a vertical profile of the magnetic field

2 $$P(z)=\{\begin{array}{ll}\quad \quad \,{e}^{-{\kappa }_{1}z} & \forall \,z > \tfrac{d}{2}\\ C\,\cosh \,{\kappa }_{2}z & \forall \,z\in [-\tfrac{h}{2},\tfrac{d}{2}]\\ \quad \quad \,\,{e}^{{\kappa }_{1}z} & \forall \,z < -\tfrac{d}{2}\end{array}$$

where $C=\tfrac{{e}^{-{\kappa }_{1}\tfrac{d}{2}}}{\cosh \,\tfrac{{\kappa }_{2}\,d}{2}}$ to ensure the continuity of the magnetic field at all interfaces. Figure 2(a) shows the profile of the magnetic field associated to the gap-plasmon propagating in a nano-gap.

The dispersion relation^1^ of the gap-plasmon is obtained using the continuity of the electric field at any interface and can be written

3 $$\frac{{\kappa }_{2}}{{\varepsilon }_{d}}\,\tanh \,({\kappa }_{2}\frac{d}{2})+\frac{{\kappa }_{1}}{{\varepsilon }_{m}}=0$$

where ${\kappa }_{1}=\sqrt{{\alpha }^{2}-{\varepsilon }_{m}{k}_{0}^{2}}$ and ${\kappa }_{2}=\sqrt{{\alpha }^{2}-{\varepsilon }_{d}{k}_{0}^{2}}$, *α* being the propagation constant in the *x* direction. Once the pulsation *ω* is fixed, the dispersion relation allows to retrieve the complex propagation constant *α* ^30^. The main characteristics of the gap-plasmon is that its effective index, defined as ${n}_{eff}=\Re (\alpha )/{k}_{0}$, diverges when the size of the gap tends to zero, as shown Fig. 2(b). This plasmonic effect occurs when light propagates more in the metal than in the dielectric. This is what allows gap-plasmon resonators to be so small compared to the wavelength in vacuum.

When the patch ends, the gap-plasmon can in fact no longer propagate. Instead, it is able to excite the surface plasmon and a waves propagating in free space^20^, but as both present very low propagation constants, the outside space represents a very high impedance mismatch for the gap-plasmon. This is of course all the more so that the gap is small and the effective index of the gap-plasmon large. As a consequence, the reflection coefficient *r* of the gap-plasmon when it encounters the edge of the patch can be very high^5, 11, 24^.

Computing this reflection coefficient is not an easy task. Its phase can be deduced from the location of the resonances themselves^5^, and more direct determinations can be difficult too^24, 31^. Finally, it can be noticed that this can be done directly using a Fourier Modal Method^32^, which relies on the computation of the modes that propagate in different vertically invariant layers to solve Maxwell’s equations and of the transmission and reflection coefficients. It directly gives access to *r* and *α*. It is even possible, relying on transformation optics techniques^33^ to introduce Perfect Matching Layers in order to get rid of the periodicity. Figure 2(c) shows the modulus of the computed reflection coefficient as a function of the waveguide width, computed using such techniques (see Methods). The phase of the reflection coefficient, which is critical for the resonance condition and thus to explain why the size of a resonator can be smaller than half of the gap-plasmon wavelength^5^, is shown on Fig. 2(c). This phase increases when the the gap closes.

**Figure 3:**
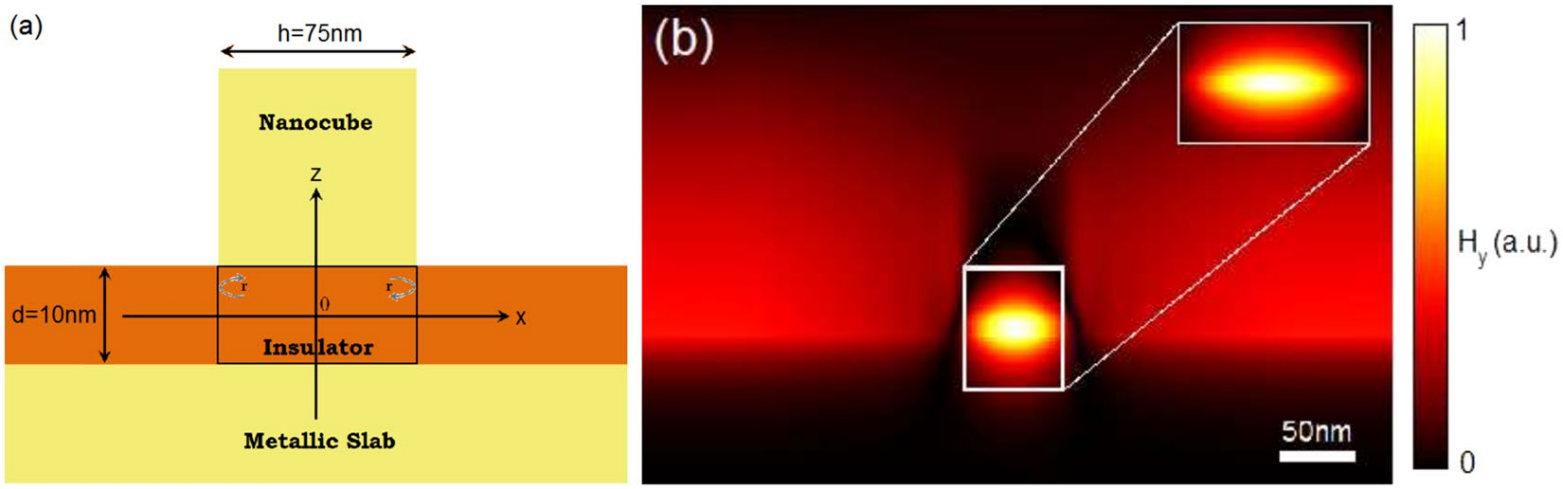
Cavity model. (**a**) Sketch representing the cavity model including the reflection coefficients of the gap-plasmon on the edges of the patch. (**b**) Map of the magnetic field (modulus) for a single 75 nm wide square patch, separated from the metallic film by a 10 nm thick dielectric layer with an optical index of 1.54 illuminated from above in normal incidence at resonance. The field profile in the region which is enclosed in the white rectangle is accurately predicted by the model (center). The inset shows the map of the magnetic field (modulus) as predicted by the cavity model when both excitation coefficients *A*_*l*_ and *A*_*r*_ are taken equal, at resonance.

Under the patch, gap-plasmons thus propagate from right to left and from left to right and are reflected on both sides of the patch, so that they form a stationary wave. This is the actual description of a cavity of size *h*, as shown on Fig. 3(a). This allows us to introduce a model for the gap-plasmon resonator, based on our knowledge of *r* and *α*, that are computed as explained above.

Since a gap-plasmon propagates along *x* towards the left and another one propagates towards the right, the amplitude of the mode, as a function of *x* under the patch can simply be written

4 $${\mathbb{A}}(x)=A\,{e}^{i\alpha x}+B\,{e}^{-i\alpha x}.$$

This means that the magnetic field associated to the stationary wave can simply be written ${H}_{y}(x,z)={\mathbb{A}}(x)P(z)$.

We choose here the edges of the cavity to be in ±$\tfrac{h}{2}$. We call *A*_*l*_ and *A*_*r*_ the amplitude with which the gap-plasmons are excited respectively on the left and on the right of the patch. We know actually very little about these quantities, except for the fact that in normal incidence, they are obviously equal: the cavity is being excited on both sides exactly the same way. In non-normal incidence, the phase difference between the two can be estimated, as the following will show, but there must be a difference in the amplitude due to the shading by the cube that is difficult to take into account. These excitations should be considered as relatively large because when a plane wave is reflected by a metallic film, the magnetic field presents a maximum on the surface. Whatever the thickness of the dielectric layer, there is always a maximum of the magnetic field at the entrance of the cavity. This explains why the cavity resonances are much more easily excited in this configuration than for dimer^34^, for instance. The resonances are, conversely, not likely to be excited *through* the patches – because they are twice as large as the skin depth.

This simple cavity model, detailed in the Supplementary Information, thus gives the amplitude of the modes in the cavity as

5 $${\mathbb{A}}(x)=[{A}_{l}\frac{{e}^{-i\alpha \tfrac{h}{2}}{e}^{i\alpha x}+r{e}^{i\alpha \tfrac{h}{2}}{e}^{-i\alpha x}}{1-{r}^{2}{e}^{2i\alpha h}}+{A}_{r}\frac{r{e}^{i\alpha \tfrac{h}{2}}{e}^{i\alpha x}+{e}^{-i\alpha \tfrac{h}{2}}{e}^{-i\alpha x}}{1-{r}^{2}{e}^{2i\alpha h}}].$$

As shown by this expression, a resonance occurs each time the quantity 1 − *r* ^2^ *e* ^2*iαh*^ presents a minimum and can be considered close enough to zero. The resonance condition, in that case, reduces to $r\simeq \pm {e}^{-i\alpha h}$. When $r\simeq {e}^{-i\alpha h}$ then the resonance is symmetric with respect to the *x* = 0 axis, which means it has an odd number of anti-nodes between −$\tfrac{d}{2}$ and $\tfrac{d}{2}$. On the contrary, when $r\simeq -{e}^{-i\alpha h}$ the magnetic field is anti-symmetric with respect to *x* = 0, and the resonance presents an even number of anti-nodes.

Keeping that in mind for later, it is then possible to get analytic expressions of all the fields, magnetic as well as electric, that are linked to the cavity and the stationary wave that exists in it - even out of resonance. When the amplitude ${\mathbb{A}}(x)$ is multiplied by the vertical profile *P*(*Z*) of the gap-plasmon, then the magnetic field ${H}_{y}(x,z)={\mathbb{A}}(x)\,P(z)$ is almost perfectly reproduced, as shown Fig. 3 where a comparison to a full simulation using a Fourier Modal Method of a single patch is shown.

Using Maxwell’s equations, the electric field can be analytically calculated (See Supplementary Information). Finally, the volumetric losses *p*(**r**) inside the metal are classically written

6 $$p({\bf{r}})=\frac{1}{2}\omega {\varepsilon }_{0}\Im ({\varepsilon }_{r})\,{\bf{E}}.{{\bf{E}}}^{\ast }.$$

As shown in the Supplementary Information, the volumetric losses can be integrated over the metallic region, to compute analytically the absorption under one patch antenna, as a function of *A*_*l*_ and *A*_*r*_

$$P={\int }_{-h/2}^{h/2}\,{\int }_{-\infty }^{+\infty }\,p({\bf{r}})\,{\rm{d}}x{\rm{d}}z.$$

## Discussion

Two different things can be done starting from here. Either use the complex expression (given in the Supplementary Information) as it is, to compute the losses in every possible situation (out of resonance, for any angle and wavelength), or to compute a simplified analytic solution that brings a better understanding but only close to resonance. Let us first assume that we are close to a resonance, with $r\simeq \pm {e}^{-i\alpha h}$. Then, as shown in the Supplementary Information, the absorption by the resonator can be written

7 $$P=\beta ({|{A}_{l}|}^{2}+{|{A}_{r}|}^{2}\pm 2\Re ({A}_{l}{A}_{r}^{\ast }))$$

In normal incidence, *A*_*l*_ = *A*_*r*_ since both ends of the cavity are excited in the same way. This explains why resonances with an even number of anti-nodes, for which *r* = −*e* ^*iαh*^ cannot be excited in normal incidence, since we have strictly *P* = 0. On the opposite, for a resonance with an odd number of anti-nodes *P* = 4*β*|*A*_*l*_|^2^ and the absorption is thus four times higher than if the cavity were excited from one side only. This shows the mechanism that is at the heart of the extraordinary large absorption cross-section that patch antenna have, when compared to their actual size. The interferometric reinforcement of the absorption, associated with the high effective index of the gap-plasmon which drives the reduction in size of the resonators, completely explains this effect.

Now it is possible to take into account the incidence angle, as long as it is not too large by simply assuming that the modulus of the excitations *A*_*r*_ and *A*_*l*_ stay close to their maximum, that is reached in normal incidence, but that the main factor driving their variation will be a slight phase shift arising from the different optical paths followed by light to reach the entrances of the gap. With a incidence angle *θ*, this phase shift is Δ*ϕ* = *k*_0_ *h* sin *θ*. We finally have an absorption at resonance that can be written either

8 $$P=2\beta {|{A}_{l}|}^{2}\,{\cos }^{2}\,(\frac{\pi \,h}{{\lambda }_{0}}\,\sin \,(\theta ))$$

for odd resonances (﻿*i.e﻿.* with an odd ﻿number﻿ of anti-nodes) and

9 $$P=2\beta {|{A}_{l}|}^{2}\,{\sin }^{2}\,(\frac{\pi \,h}{{\lambda }_{0}}\,\sin \,(\theta ))$$

for even resonances (*﻿i.e.* with ﻿a﻿n even number of anti-nodes). Changing the angle of incidence thus lowers the absorption of odd resonances, while allowing the even resonances to be excited and to appear in the spectrum.

**Figure 4:**
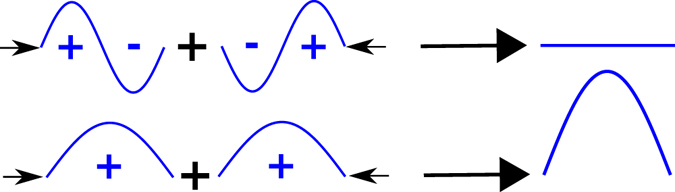
The interferometric control of the absorption in patch antennas summarized in normal incidence: adding two anti-symmetric modes excited from left and right leads to a complete cancellation, while adding even modes leads to an enhancement. For oblique incidence, this control is lowered, so that the even modes don’t completely cancel each other, and the odd modes are less enhanced.

Our findings can be summarized by a simple scheme (see Fig. 4). Odd (resp. even) resonances are actually symmetric (resp. anti-symmetric). Since each side of the patch is excited by the incoming field, the field under the patch can be seen as a superposition of two modes: one excited from the left and one excited from the right. In normal incidence, both excitations are perfectly in phase. When the resonance should be odd (and symmetric), then the two modes add up so that the amplitude of the field is exactly twice what it would be if the resonance had been excited from one side only (and the absorption is multiplied by four). When the resonances should be even (and anti-symmetric) the two modes interfere destructively, and the resonance is completely canceled. For oblique incidence, the two excitations are not in phase anymore, disturbing the interference between the modes. Odd resonances see their efficiency decrease, while even resonances can be excited.

**Figure 5:**
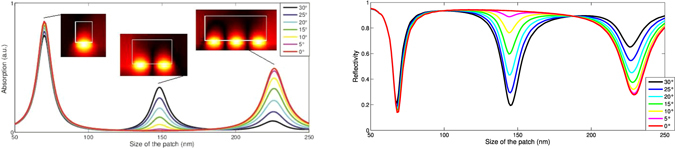
Absorption spectrum for a silver nanopatch of variable width (in nm) from the model (left) and from the reference code (right). The layer is 5 nm thick, the wavelength 950 nm.

Now we can use the model described above, without any assumption on *r*, out of the resonance condition to try to predict, as the incidence angle increases, how the absorption linked to the resonance will evolve. Using the general expression for the absorption obtained thanks to our model, we plot the absorption as a function of the patch width (see Fig. 5) and compare it to the reflectance of the structure for different incidence angles . The accuracy of the model can be clearly seen. It is almost perfect in normal incidence and for large angles the variation of the interferometric effect is not perfectly reproduced - probably because our assumptions regarding the optical path and the shading are not fully accurate. However, this definitely validates our physical interpretation of the nanopatch optical response.

We underline that the destructive interference that literally kills odd resonances in normal incidence can be utilized to assess whether the angle of incidence is actually zero or not. As soon as the incidence angle differs from zero, the interferometric control will stop and the absorption linked to the resonance will sharply increase, as shown on the spectrum. This would be the optical equivalent of an optical set square at a nanometric level.

## Conclusion

We have shown that a optical patch antenna can be considered as a Fabry-Perot cavity excited from both sides and that this explains why the absorption by even resonances is reinforced by an interferometric control effect while odd resonances are completely tamed by the same effect. Previous authors have already evoked the symmetry mismatch between the incident wave and the resonances^24, 31^, but the interferometric control pictures offers a way to quantify this effect. Using an analytic model whose parameters are computed numerically, we have been able to show how this effect varies when the incidence angle is changed, allowing odd resonances to be excited and slightly lowering the absorption efficiency of even resonances. This gives a better understanding of why the absorption of metasurfaces made with such resonators decreases when the angle of incidence increases - and shows that modifying the angle of incidence is an actual way to control the absorption cross section of each individual resonator and thus light concentration of the fluorescence enhancement for instance^18^. We think that having in mind this fundamental mechanism of the absorption by optical patch antennas will help find new applications or new techniques. It may help to forecast the radiation pattern of film-coupled nanocubes^18^, and shows that the angle of incidence is a way to control the absorption cross-section of a single patch. Finally, the fact that the excitation of odd resonances is strictly impossible at normal incidence could well be utilized to fabricate an optical set-square, giving an immediate application to the interferometric control of the absorption by optical patch antennas.

## Methods

We used a Fourier Modal Method with coordinate stretching and Perfect Matching Layers^33^ to first solve the modal problem in a metal-insulator-metal structure. The period is of 930 nm with a 200 nm thick PML and a stretching parameter *η* = 0.99. On one side, we have 120 nm of metal and 600 nm on the other side. We take the mode with the smallest imaginary part for the propagation constant. This is the gap plasmon.

We then compute the scattering matrix when the above structure is put next to a structure with the same parameters, except that the 600 nm metallic part is replaced by air. The rest of the parameters are kept the same. The mode with the lowest imaginary part for its propagation constant is then the surface plasmon. Using the boundary conditions allows to access the scattering matrix of the problem and then retrieve the coefficient of the scattering matrix corresponding to an incoming and outgoing gap-plasmon. The coefficient constitutes the reflection coefficient of the gap-plasmon on each edge of the structure.

## Electronic supplementary material

Supplementary Information
