# Supplementary material for: Interferometric control of the absorption in optical patch antennas
Source: Sci Rep. 2017 Jun 7;7:2941. doi: 10.1038/s41598-017-03064-6 (PMC5462803; doi:10.1038/s41598-017-03064-6)
Supplement: Supplementary file 1 — Supplementary Information [file 41598_2017_3064_MOESM1_ESM.pdf]

Interferometric control of the absorption in optical patch antennas: Supplementary Information

Caroline Lemaître[1], Emmanuel Centeno[1], and Antoine Moreau[1*]

[1]Université Clermont Auvergne, CNRS, Institut Pascal, F-63000 CLERMONT-FERRAND, FRANCE
[*]antoine.moreau@uca.fr

ABSTRACT

This document contains supplementary informations, backing the article entitled ”Interferometric control of the absorption in optical patch antennas”.

A Supplementary Equations

A.1 Cavity model

The field inside a horizontal Fabry-Perot cavity excited from the left with an amplitude A_r and from the right with an amplitude A_r can be obtained by introducing a simple model. We assume that the wave excited in the cavity is reflected in $x = -\frac{h}{2}$ and in $x = \frac{h}{2}$ with a reflection coefficient r. We call A the amplitude of the wave towards the right in $x = 0$, while B is the amplitude towards the left in $x = 0$. The amplitude inside the cavity, whatever the kind of wave that is excited, can be written

$$\mathbb{A}(x) = A\,e^{i\alpha x} + B\,e^{-i\alpha x}.$$

If we then write that the amplitude of the wave in $x = -\frac{h}{2}$ propagating towards the right is the result of the reflection of the wave coming from the left with an amplitude $Be^{i\alpha\frac{h}{2}}$ and of the excitation A_l, then we can write that

$$Ae^{-i\alpha\frac{h}{2}} = A_l + rBe^{i\alpha\frac{h}{2}}.$$

We can do the same reasoning for the right edge of the patch and write

$$Be^{-i\alpha\frac{h}{2}} = A_r + rAe^{i\alpha\frac{h}{2}}.$$

Using this equations in the left edge and the right edge, we find :

$$\begin{cases} A = A_l e^{i\alpha\frac{h}{2}} + rBe^{i\alpha h} \\ B = A_r e^{i\alpha\frac{h}{2}} + rAe^{i\alpha h} \end{cases}$$

$$\begin{cases} A = A_l e^{i\alpha\frac{h}{2}} + rA_r e^{3i\alpha\frac{h}{2}} + r^2 Ae^{2i\alpha h} \\ A = A_r e^{i\alpha\frac{h}{2}} + rA_l e^{3i\alpha\frac{h}{2}} + r^2 Be^{2i\alpha h} \end{cases}$$

$$\begin{cases} A(1 - r^2 e^{2i\alpha h}) = e^{i\alpha h}(A_l e^{-i\alpha\frac{h}{2}} + rA_r e^{i\alpha\frac{h}{2}}) \\ B(1 - r^2 e^{2i\alpha h}) = e^{i\alpha h}(A_r e^{-i\alpha\frac{h}{2}} + rA_l e^{i\alpha\frac{h}{2}}) \end{cases}$$

$$\begin{cases} A = te^{i\alpha h}\dfrac{A_l e^{-i\alpha\frac{h}{2}} + rA_r e^{i\alpha\frac{h}{2}}}{1 - r^2 e^{2i\alpha h}} \\ \\ B = te^{i\alpha h}\dfrac{A_r e^{-i\alpha\frac{h}{2}} + rA_l e^{i\alpha\frac{h}{2}}}{1 - r^2 e^{2i\alpha h}} \end{cases}$$

Finally, the amplitude $\mathbb{A}(x)$ can be written

$$\mathbb{A}(x) = e^{i\alpha h}\left\{\left[\frac{A_l e^{-i\alpha\frac{h}{2}} + rA_r e^{i\alpha\frac{h}{2}}}{1-r^2e^{2i\alpha h}}\right]e^{i\alpha x} + \left[\frac{A_r e^{-i\alpha\frac{h}{2}} + rA_l e^{i\alpha\frac{h}{2}}}{1-r^2e^{2i\alpha h}}\right]e^{-i\alpha x}\right\}$$

or

$$\mathbb{A}(x) = \left[A'_l\frac{e^{-i\alpha\frac{h}{2}}e^{i\alpha x} + re^{i\alpha\frac{h}{2}}e^{-i\alpha x}}{1-r^2e^{2i\alpha h}} + A'_r\frac{re^{i\alpha\frac{h}{2}}e^{i\alpha x} + e^{-i\alpha\frac{h}{2}}e^{-i\alpha x}}{1-r^2e^{2i\alpha h}}\right]$$

A.2 Magnetic field inside the resonator

By multiplying this field by the profile of the gap-plasmon alog the z axis, we can write the field H_y. In the following we thus present the fields in three parts. The upper part is the field inside the patch, the central part is the field in the spacer and the lower part is the field inside the metallic substrate. This gives

$$\begin{cases} H_y &= \dfrac{1}{1-r^2e^{2i\alpha h}}\left[A'_l\left(e^{-i\alpha\frac{h}{2}}e^{i\alpha x} + re^{i\alpha\frac{h}{2}}e^{-i\alpha x}\right) + A'_r\left(re^{i\alpha\frac{h}{2}}e^{i\alpha x} + e^{-i\alpha\frac{h}{2}}e^{-i\alpha x}\right)\right]Ae^{-\kappa_1 z} \\ H_y &= \dfrac{1}{1-r^2e^{2i\alpha h}}\left[A'_l\left(e^{-i\alpha\frac{h}{2}}e^{i\alpha x} + re^{i\alpha\frac{h}{2}}e^{-i\alpha x}\right) + A'_r\left(re^{i\alpha\frac{h}{2}}e^{i\alpha x} + e^{-i\alpha\frac{h}{2}}e^{-i\alpha x}\right)\right]B\cosh(\kappa_2 z) \\ H_y &= \dfrac{1}{1-r^2e^{2i\alpha h}}\left[A'_l\left(e^{-i\alpha\frac{h}{2}}e^{i\alpha x} + re^{i\alpha\frac{h}{2}}e^{-i\alpha x}\right) + A'_r\left(re^{i\alpha\frac{h}{2}}e^{i\alpha x} + e^{-i\alpha\frac{h}{2}}e^{-i\alpha x}\right)\right]Ae^{\kappa_1 z} \end{cases}$$

A.3 Electric field inside the resonator

Using Maxwell's equations, for TM polarization

$$E_x = -\frac{i}{\omega\varepsilon_0\varepsilon_r}\frac{\partial H_y}{\partial z}$$

$$E_z = \frac{i}{\omega\varepsilon_0\varepsilon_r}\frac{\partial H_y}{\partial x},$$

it is easy to obtain the fields E_x and E_z by deriving the field H_y with respect to z and x

The fields are presented in three parts just like for the magnetic field. We get

$$\begin{cases} E_x &= \dfrac{i}{\omega\varepsilon_0\varepsilon_r}\dfrac{1}{1-r^2e^{2i\alpha h}}\left[A'_l\left(e^{-i\alpha\frac{h}{2}}e^{i\alpha x} + re^{i\alpha\frac{h}{2}}e^{-i\alpha x}\right) + A'_r\left(re^{i\alpha\frac{h}{2}}e^{i\alpha x} + e^{-i\alpha\frac{h}{2}}e^{-i\alpha x}\right)\right]\kappa_1 Ae^{-\kappa_1 z} \\ E_x &= -\dfrac{i}{\omega\varepsilon_0\varepsilon_r}\dfrac{1}{1-r^2e^{2i\alpha h}}\left[A'_l\left(e^{-i\alpha\frac{h}{2}}e^{i\alpha x} + re^{i\alpha\frac{h}{2}}e^{-i\alpha x}\right) + A'_r\left(re^{i\alpha\frac{h}{2}}e^{i\alpha x} + e^{-i\alpha\frac{h}{2}}e^{-i\alpha x}\right)\right]\kappa_2 B\sinh(\kappa_2 z) \\ E_x &= -\dfrac{i}{\omega\varepsilon_0\varepsilon_r}\dfrac{1}{1-r^2e^{2i\alpha h}}\left[A'_l\left(e^{-i\alpha\frac{h}{2}}e^{i\alpha x} + re^{i\alpha\frac{h}{2}}e^{-i\alpha x}\right) + A'_r\left(re^{i\alpha\frac{h}{2}}e^{i\alpha x} + e^{-i\alpha\frac{h}{2}}e^{-i\alpha x}\right)\right]\kappa_1 Ae^{\kappa_1 z} \end{cases}$$

$$\begin{cases} E_z &= \dfrac{i}{\omega\varepsilon_0\varepsilon_r}\dfrac{1}{1-r^2e^{2i\alpha h}}\left[A'_l\left(e^{-i\alpha\frac{h}{2}}e^{i\alpha x} - re^{i\alpha\frac{h}{2}}e^{-i\alpha x}\right) + A'_r\left(re^{i\alpha\frac{h}{2}}e^{i\alpha x} - e^{-i\alpha\frac{h}{2}}e^{-i\alpha x}\right)\right]i\alpha Ae^{-\kappa_1 z} \\ E_z &= \dfrac{i}{\omega\varepsilon_0\varepsilon_r}\dfrac{1}{1-r^2e^{2i\alpha h}}\left[A'_l\left(e^{-i\alpha\frac{h}{2}}e^{i\alpha x} - re^{i\alpha\frac{h}{2}}e^{-i\alpha x}\right) + A'_r\left(re^{i\alpha\frac{h}{2}}e^{i\alpha x} - e^{-i\alpha\frac{h}{2}}e^{-i\alpha x}\right)\right]i\alpha B\cosh(\kappa_2 z) \\ E_z &= \dfrac{i}{\omega\varepsilon_0\varepsilon_r}\dfrac{1}{1-r^2e^{2i\alpha h}}\left[A'_l\left(e^{-i\alpha\frac{h}{2}}e^{i\alpha x} - re^{i\alpha\frac{h}{2}}e^{-i\alpha x}\right) + A'_r\left(re^{i\alpha\frac{h}{2}}e^{i\alpha x} - e^{-i\alpha\frac{h}{2}}e^{-i\alpha x}\right)\right]i\alpha Ae^{\kappa_1 z} \end{cases}$$

A.4 Losses

The volumic losses are simply given by

$$p(\mathbf{r}) = \frac{1}{2}\omega\varepsilon_0\Im(\varepsilon_r)\mathbf{E}.\mathbf{E}^*.$$

which shows they can only take place in the metal. The expression for the volumic losses can be obtained in the patch (upper part of the waveguide). Given the symmetry of the problem, simply replacing $e^{-\kappa_1 z}$ with $e^{\kappa_1 z}$ in the following expressions gives the volumic losses in the metallic substrate. In the patch, we get

$$||\vec{E}||^2 = E_x E_x^* + E_z E_z^*$$

$$\begin{aligned}
E_x E_x^* =& \frac{1}{\omega^2 \varepsilon_0^2 \varepsilon_r^2} \frac{e^{-2\Im(\alpha)h}}{1 - r^2 e^{2i\alpha h} - r^{*2} e^{-2i\alpha^* h} + r^2 r^{*2} e^{-4\Im(\alpha)h}} |\kappa_2|^2 |A|^2 e^{-\kappa_1 z} \times \\
& \Big\{ |A_l|^2 \left(e^{\Im(\alpha)h} e^{-2\Im(\alpha)x} + r e^{i\Re(\alpha)h} e^{-2i\Re(\alpha)x} + r^* e^{-i\Re(\alpha)h} e^{2i\Re(\alpha)x} + |r|^2 e^{-\Im(\alpha)h} e^{2\Im(\alpha)x} \right) \\
& + |A_r|^2 \left(e^{\Im(\alpha)h} e^{2\Im(\alpha)x} + r e^{i\Re(\alpha)h} e^{2i\Re(\alpha)x} + r^* e^{-i\Re(\alpha)h} e^{-2i\Re(\alpha)x} + |r|^2 e^{-\Im(\alpha)h} e^{-2\Im(\alpha)x} \right) \\
& + A_l A_r^* \left(e^{\Im(\alpha)h} e^{2i\Re(\alpha)x} + r e^{i\Re(\alpha)h} e^{2\Im(\alpha)x} + r^* e^{-i\Re(\alpha)h} e^{-2\Im(\alpha)x} + |r|^2 e^{-\Im(\alpha)h} e^{-2i\Re(\alpha)x} \right) \\
& + A_l^* A_r \left(e^{\Im(\alpha)h} e^{-2i\Re(\alpha)x} + r e^{i\Re(\alpha)h} e^{-2\Im(\alpha)x} + r^* e^{-i\Re(\alpha)h} e^{2\Im(\alpha)x} + |r|^2 e^{-\Im(\alpha)h} e^{2i\Re(\alpha)x} \right) \Big\}
\end{aligned}$$

$$\begin{aligned}
E_z E_z^* =& \frac{1}{\omega^2 \varepsilon_0^2 \varepsilon_r^2} \frac{e^{-2\Im(\alpha)h}}{1 - r^2 e^{2i\alpha h} - r^{*2} e^{-2i\alpha^* h} + r^2 r^{*2} e^{-4\Im(\alpha)h}} |\alpha|^2 |A|^2 e^{-\kappa_1 z} \times \\
& \Big\{ |A_l|^2 \left(e^{\Im(\alpha)h} e^{-2\Im(\alpha)x} - r e^{i\Re(\alpha)h} e^{-2i\Re(\alpha)x} - r^* e^{-i\Re(\alpha)h} e^{2i\Re(\alpha)x} + |r|^2 e^{-\Im(\alpha)h} e^{2\Im(\alpha)x} \right) \\
& + |A_r|^2 \left(e^{\Im(\alpha)h} e^{2\Im(\alpha)x} - r e^{i\Re(\alpha)h} e^{2i\Re(\alpha)x} - r^* e^{-i\Re(\alpha)h} e^{-2i\Re(\alpha)x} + |r|^2 e^{-\Im(\alpha)h} e^{-2\Im(\alpha)x} \right) \\
& + A_l A_r^* \left(-e^{\Im(\alpha)h} e^{2i\Re(\alpha)x} + r e^{i\Re(\alpha)h} e^{2\Im(\alpha)x} + r^* e^{-i\Re(\alpha)h} e^{-2\Im(\alpha)x} - |r|^2 e^{-\Im(\alpha)h} e^{-2i\Re(\alpha)x} \right) \\
& + A_l^* A_r \left(-e^{\Im(\alpha)h} e^{-2i\Re(\alpha)x} + r e^{i\Re(\alpha)h} e^{-2\Im(\alpha)x} + r^* e^{-i\Re(\alpha)h} e^{2\Im(\alpha)x} - |r|^2 e^{-\Im(\alpha)h} e^{2i\Re(\alpha)x} \right) \Big\}
\end{aligned}$$

$$\begin{aligned}
p(\mathbf{r}) =& \frac{1}{2} \Re \Bigg\{ \frac{-i\left(\varepsilon_0 \varepsilon_r - \varepsilon_0\right)}{\omega \varepsilon_0^2 \varepsilon_r^2} \frac{e^{-2\Im(\alpha)h}}{1 - r^2 e^{2i\alpha h} - r^{*2} e^{-2i\alpha^* h} + r^2 r^{*2} e^{-4\Im(\alpha)h}} |A|^2 e^{-2\Re(\kappa_1)z} \times \\
& \Big\{ \left(|\kappa_2|^2 + |\alpha|^2 \right) \times \Big[|A_l|^2 \left(e^{\Im(\alpha)h} e^{-2\Im(\alpha)x} + |r|^2 e^{-\Im(\alpha)h} e^{2\Im(\alpha)x} \right) \\
& \quad + |A_r|^2 \left(e^{\Im(\alpha)h} e^{2\Im(\alpha)x} + |r|^2 e^{-\Im(\alpha)h} e^{-2\Im(\alpha)x} \right) \\
& \quad + A_l A_r^* \left(r e^{i\Re(\alpha)h} e^{2\Im(\alpha)x} + r^* e^{-i\Re(\alpha)h} e^{-2\Im(\alpha)x} \right) \\
& \quad + A_l^* A_r \left(r e^{i\Re(\alpha)h} e^{-2\Im(\alpha)x} + r^* e^{-i\Re(\alpha)h} e^{2\Im(\alpha)x} \right) \Big] \\
& + \left(|\kappa_2|^2 - |\alpha|^2 \right) \times \Big[|A_l|^2 \left(r e^{i\Re(\alpha)h} e^{-2i\Re(\alpha)x} + r^* e^{-i\Re(\alpha)h} e^{2i\Re(\alpha)x} \right) \\
& \quad + |A_r|^2 \left(r e^{i\Re(\alpha)h} e^{2i\Re(\alpha)x} + r^* e^{-i\Re(\alpha)h} e^{-2i\Re(\alpha)x} \right) \\
& \quad + A_l A_r^* \left(e^{\Im(\alpha)h} e^{2i\Re(\alpha)x} + |r|^2 e^{-\Im(\alpha)h} e^{-2i\Re(\alpha)x} \right) \\
& \quad + A_l^* A_r \left(e^{\Im(\alpha)h} e^{-2i\Re(\alpha)x} + |r|^2 e^{-\Im(\alpha)h} e^{2i\Re(\alpha)x} \right) \Big] \Big\} \Bigg\}
\end{aligned}$$

The imaginary part of α is usually negligible when compared to its real part. For instance, the effective index of the gap-plasmon for a 10 nm gap is $2.728918+0.024629i$. There are two orders of magnitude between the imaginary and the real part. So we consider α as being real and the losses become

$$p(\mathbf{r}) = \frac{1}{2}\Re\left\{ \frac{-i\left(\varepsilon_0\varepsilon_r-\varepsilon_0\right)}{\omega\varepsilon_0^2\varepsilon_r^2} \frac{1}{1-r^2e^{2i\alpha h}-r^{*2}e^{-2i\alpha h}+r^2r^{*2}} |A|^2 e^{-2\Re(\kappa_1)z} \times \left\{\left(|\kappa_2|^2+\alpha^2\right)\times \right.\right.$$
$$\left[|A_l|^2\left(1+|r|^2\right)+|A_r|^2\left(1+|r|^2\right)+A_lA_r^*\left(re^{i\alpha h}+r^*e^{-i\alpha h}\right)+A_l^*A_r\left(re^{i\alpha h}+r^*e^{-i\alpha h}\right)\right]$$
$$+\left(|\kappa_2|^2-\alpha^2\right)\times\left[|A_l|^2\left(re^{i\alpha h}e^{-2i\alpha x}+r^*e^{-i\alpha h}e^{2i\alpha x}\right)+|A_r|^2\left(re^{i\alpha h}e^{2i\alpha x}+r^*e^{-i\alpha h}e^{-2i\alpha x}\right)\right.$$
$$\left.\left.\left.+A_lA_r^*\left(e^{2i\alpha x}+|r|^2e^{-2i\alpha x}\right)+A_l^*A_r\left(e^{-2i\alpha x}+|r|^2e^{2i\alpha x}\right)\right]\right\}\right\}$$

Computing the losses linked to the resonance require that we now integrate over all the space :

$$P=\int_{-\frac{h}{2}}^{\frac{h}{2}}\int_{\frac{d}{2}}^{\infty}p(\mathbf{r})dzdx+\int_{-\frac{h}{2}}^{\frac{h}{2}}\int_{-\infty}^{-\frac{d}{2}}p(\mathbf{r})dzdx$$

$$\int_{-\frac{h}{2}}^{\frac{h}{2}}\int_{\frac{d}{2}}^{\infty}p(\mathbf{r})dzdx =$$

$$-\frac{1}{2}\Re\left\{\frac{-i\left(\varepsilon_0\varepsilon_r-\varepsilon_0\right)}{\omega\varepsilon_0^2\varepsilon_r^2}\frac{1}{1-r^2e^{2i\alpha h}-r^{*2}e^{-2i\alpha h}+r^2r^{*2}}|A|^2e^{-\Re(\kappa_1)d}\times\right.$$

$$\left\{|A_l|^2\left[\left(|\kappa_2|^2+\alpha^2\right)\left(h+|r|^2h\right)+\frac{|\kappa_2|^2-\alpha^2}{2i\alpha}\left(r^*-r+re^{2i\alpha h}-r^*e^{-2i\alpha h}\right)\right]\right.$$

$$|A_r|^2\left[\left(|\kappa_2|^2+\alpha^2\right)\left(h+|r|^2h\right)+\frac{|\kappa_2|^2-\alpha^2}{2i\alpha}\left(r^*-r+re^{2i\alpha h}-r^*e^{-2i\alpha h}\right)\right]$$

$$A_lA_r^*\left[\left(|\kappa_2|^2+\alpha^2\right)\left(hre^{i\alpha h}+hr^*e^{-i\alpha h}\right)+\frac{|\kappa_2|^2-\alpha^2}{2i\alpha}\left(e^{i\alpha h}-e^{-i\alpha h}+|r|^2e^{i\alpha h}-|r|^2e^{-i\alpha h}\right)\right]$$

$$\left.\left.A_l^*A_r\left[\left(|\kappa_2|^2+\alpha^2\right)\left(hre^{i\alpha h}+hr^*e^{-i\alpha h}\right)+\frac{|\kappa_2|^2-\alpha^2}{2i\alpha}\left(e^{i\alpha h}-e^{-i\alpha h}+|r|^2e^{i\alpha h}-|r|^2e^{-i\alpha h}\right)\right]\right\}\right\}$$

The above expression has been used to model the absorption of a nano-patch antenna in the article. Now if we want to reach a more understandable expression, some supplementary assumptions, that will make more obvious what happens in the complex expression above. As already mentioned above, close enough to a resonance, we can consider that we have $1-r^2e^{2i\alpha h}\simeq 0$ which means that we can write $r=\pm e^{-i\alpha h}$. The plus or minus (respectively) sign depends on whether the resonance has an odd or even number of anti-nodes (respectively). This means that we can assume, in the above expression, that $|r|^2\approx 1$. Using these

approximations we get

$$\int_{-\frac{h}{2}}^{\frac{h}{2}}\int_{\frac{d}{2}}^{\infty} p(\mathbf{r})dzdx = -\Re\left\{\frac{-i\left(\varepsilon_0\varepsilon_r-\varepsilon_0\right)}{\omega\varepsilon_0^2\varepsilon_r^2}\frac{1}{1-r^2e^{2i\alpha h}-r^{*2}e^{-2i\alpha h}+r^2r^{*2}}|A|^2e^{-\Re(\kappa_1)d}\times\right.$$

$$\left\{|A_l|^2\left[\left(|\kappa_2|^2+\alpha^2\right)h+\frac{|\kappa_2|^2-\alpha^2}{2i\alpha}\left(e^{i\alpha h}-e^{-i\alpha h}\right)\right]\right.$$

$$+|A_r|^2\left[\left(|\kappa_2|^2+\alpha^2\right)h+\frac{|\kappa_2|^2-\alpha^2}{2i\alpha}\left(e^{i\alpha h}-e^{-i\alpha h}\right)\right]$$

$$\left.\left.\pm\left(A_lA_r^*+A_l^*A_r\right)\left[\left(|\kappa_2|^2+\alpha^2\right)h+\frac{|\kappa_2|^2-\alpha^2}{2i\alpha}\left(e^{i\alpha h}-e^{-i\alpha h}\right)\right]\right\}\right\}$$

That is to say :

$$P=\beta|A_l|^2+\beta|A_r|^2\pm\beta\left(A_lA_r^*+A_l^*A_r\right)$$

with

$$\beta=-\Re\left\{\frac{-i\left(\varepsilon_0\varepsilon_r-\varepsilon_0\right)}{\omega\varepsilon_0^2\varepsilon_r^2}\frac{1}{1-r^2e^{2i\alpha h}-r^{*2}e^{-2i\alpha h}+r^2r^{*2}}e^{-\Re(\kappa_1)d}\left[\left(|\kappa_2|^2+\alpha^2\right)h+\frac{|\kappa_2|^2-\alpha^2}{\alpha}\sin\alpha h\right]\right\}$$

B Supplementary Figure

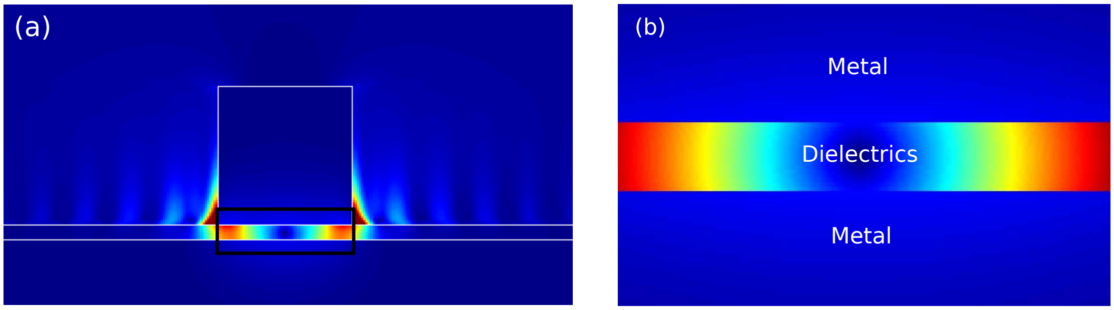

Figure S1. (a) Map of the modulus of the total electric field, obtained using a Fourier Modal Method. The width of the patch is 75 nm and the thickness of the spacer is 10 nm. (b) Map of the modulus of the electric field, obtained using analytic expressions for the E_x and E_z fields. This map (75 nm wide) must be compared with the electric field enclosed in the black region (left).
